# Nanobiotechnology and Immunotherapy: Two Powerful and Cooperative Allies against Cancer

**DOI:** 10.3390/cancers13153765

**Published:** 2021-07-27

**Authors:** Francesco Mainini, Francesca De Santis, Giovanni Fucà, Massimo Di Nicola, Licia Rivoltini, Michael Eccles

**Affiliations:** 1 Immunotherapy and Innovative Therapeutics Unit, Department of Medical Oncology, Fondazione IRCCS Istituto Nazionale dei Tumori, 20133 Milan, Italy; francesco.mainini@gmail.com (F.M.); Francesca.DeSantis@istitutotumori.mi.it (F.D.S.); Giovanni.Fuca@istitutotumori.mi.it (G.F.); Massimo.DiNicola@istitutotumori.mi.it (M.D.N.); 2 Unit of Immunotherapy of Human Tumors, Fondazione IRCCS Istituto Nazionale dei Tumori, 20133 Milan, Italy; licia.rivoltini@istitutotumori.mi.it; 3 Department of Pathology, Dunedin School of Medicine, University of Otago, Dunedin 9054, New Zealand

**Keywords:** nanoparticles, nanomedicine, immunotherapy, adoptive cell therapy, nanovaccines, immunomodulation, tumor microenvironment, nanotechnology, immune checkpoint, PD-1

## Abstract

**Simple Summary:**

Conventional anti-cancer treatments for metastatic tumors include chemotherapy and radiation. These approaches can result in harmful side-effects and, in the vast majority of cases, are not curative. Recently, novel treatments have been developed in order to stimulate the host immune system to fight cancer. This type of therapeutic approach, called immunotherapy, has gained a lot of attention in recent years due to discoveries that have deciphered the immunosuppressive role of the tumor microenvironment and underpinning molecular signals. To enhance the delivery of therapeutic drugs to the tumor site, nanoparticle-based delivery systems can be used to reduce off-target effects, and to modulate immune cells present in the tumor microenvironment. This novel therapeutic approach can synergize with other immunotherapies such as immune checkpoint blockade inhibitors and adoptive cell therapy, by enhancing the infiltration of activated immune cells to the tumor site, and by limiting local immunosuppression.

**Abstract:**

A number of novel cancer therapies have recently emerged that have rapidly moved from the bench to the clinic. Onco-immunotherapies, such as immune checkpoint blockade inhibitors and adoptive cell therapies, have revolutionized the field, since they provide a way to induce strong anti-tumor immune responses, which are able to fight cancer effectively. However, despite showing great efficacy in hematological and some solid tumors, unresponsiveness, development of therapy resistance and the development of serious adverse effects, limit their capacity to impact the vast majority of tumors. Nanoparticle-based delivery systems are versatile vehicles for a wide variety of molecular cargoes and provide an innovative strategy to improve conventional onco-immunotherapies. They can be finely tuned to release their contents in the tumor microenvironment, or to deliver combinations of adjuvants and antigens in the case of nanovaccines. In this review, we summarize the recent advancements in the field of nanobiotechnology, to remodel the tumor microenvironment and to enhance immunotherapies.

## 1. Introduction

The tumor microenvironment (TME) is a complex system composed of proliferating tumor cells, infiltrating immune cells, the extracellular matrix (ECM), blood vessels and a variety of associated cells. The multifaceted cellular compartments present in the TME cooperate in the maintenance of the necessary conditions for tumor development: (1) angiogenesis, to offer nutritional support for tumor growth, and (2) immunosuppression, to inhibit the adaptive immune response against cancer cells [1]. In particular, tumor infiltrating immune cells not only fail to exercise their anti-tumor effector function, but they are able to promote tumor growth, invasion and metastasis [2].

Recently, investigation of the molecular mechanisms behind the immunosuppressive state in the TME led to the discovery of immune checkpoint inhibitors (ICIs), which changed the paradigm of cancer treatment, giving rise to novel immunotherapeutic options able to induce a strong infiltration of active immune cells in the TME, with consequent control of tumor growth [3]. ICIs currently used in the clinical setting are monoclonal antibodies (mAb) able to block the activity of cytotoxic T-lymphocyte antigen-4 (CTLA-4) or programmed cell death protein 1 (PD-1), expressed by T cells. Both CTLA-4 and PD-1 are repressor molecules that de-activate T effector function [4]. These checkpoint proteins are essential to control the balance between self-tolerance and auto-immunity [5]. Currently, the anti CTL4 mAb, Ipilumab, is approved for the treatment of unresectable melanoma, advanced renal cell carcinoma and advanced colorectal cancer in combination with the anti PD-1 mAb Nivolumab [6,7,8]. Interestingly, Nivolumab is active as standalone treatment in melanoma and non-small cell lung carcinoma (NSCLC) [9]. Pembrolizumab is another anti PD-1 mAb, employed for the treatment of a wide variety of cancer types [10]. Overall, ICIs are particularly effective for the treatment of high mutational burden, mismatch repair-deficient or high microsatellite instability tumors, where many mutations are present, thus favoring the generation of anti-tumor immune responses against specific tumor associated neo-antigens [10].

Another type of novel immunotherapeutic treatment is adoptive cellular transfer (ACT). In this case, patient-derived immune cells are expanded ex vivo and re-infused into the body. ACT-based cancer immunotherapy treatments mainly involve the re-infusion of genetically modified T cells [11]. However, other cell types such as natural killer cells (NK) and macrophages have been explored [12,13]. T cell–based ACT can be divided into three sub-categories: (1) tumor-infiltrating lymphocytes (TILs), where patient derived T cells are simply expanded and re-infused; (2) T cell receptor (TCR) engineered cells, where a TCR that is able to identify a specific tumor antigen, is added into the genome of T cells; and (3) chimeric antigen receptors T cells (CAR-T), where T cells are modified with a single chain variable fragment (scFv) able to recognize neo-antigen epitopes in a major histocompatibility complex (MHC) independent manner [14].

Despite the large success of ICIs and ACT in hematological cancers, their effectiveness in solid tumors remains limited due to acquired resistance to therapy and evasion of anti-tumor immunity [15]. Resistance to immunotherapy is caused by many factors including upregulation of immune checkpoints in the TME, downregulation of MHC molecules in tumor cells, loss of target antigens and secretion of immune suppressive signals by tumor-associated macrophages (TAMs), myeloid-derived suppressor cells (MDSCs) and T regulatory cells (Tregs) [15].

To enhance the impact of immunotherapies in solid tumors, multiple therapeutic strategies could be employed simultaneously to effectively attack cancer cells, while at the same time reducing the immunosuppressive molecular signals in the TME. For example, standard treatments (chemotherapy and radiation) can be combined effectively with ICIs and ACT to reduce immunosuppressive cells in the TME, and enhance immunotherapy [16]. Other novel approaches comprise the use of nanoparticles (NP) to deliver immunomodulatory molecules to the TME or to further boost the anti-tumor immune response in the case of cancer nanovaccines [17,18].

Interestingly, in the vast majority of advanced tumors, the TME is characterized by acidosis and hypoxia [19]. These two characteristics derive from the altered metabolism of cancer cells, fueled by an enhanced glycolytic activity necessary to support active cell proliferation [20]. Glycolysis results in the production of lactic acid, which is excreted in the TME by cancer cells, causing acidification of the TME. On the other hand, a hypoxic TME is caused by aberrant vascularization and poor blood supply [21]. Tumors tend to become hypoxic as a consequence of their growth, which leads to a lower blood supply to the inner part of the tumor. This can give rise to necrosis and a perpetually inflamed state in tumors, which was initially described more than thirty years ago, as a “wound that does not heal” [22].

NP-based delivery systems can be designed to take advantage of the aberrant vasculature, the acidic or hypoxic TME, to induce the release of therapeutic drugs directly in the TME, reducing off-target side effects [23]. In the last twenty years, the discovery of novel biomaterials has dramatically impacted on the field of nanobiotechnology, such as, for example, the addition of novel stimuli-responsive polymers, which can be used to develop advanced nanostructures with the ability to improve the pharmacokinetic properties of many drugs used in oncology [24]. The application of nanotherapeutics to cancer therapy has already reached the clinical stage, with more than ten FDA-approved nanoformulations, mainly employed for the delivery of chemotherapeutics such as doxorubicin (DOX), daunorubicin, paclitaxel and irinotecan, among others [25]. In addition, nanovaccines designed for the co-delivery of antigen and adjuvants to antigen presenting cells (APCs), have also been recently deployed for COVID-19, opening novel avenues for the use of nucleic acids-loaded NP for cancer therapy in the near future [26].

In this review, we summarize recent advances in the field of nanobiotechnology applied to cancer therapy with a specific focus on the immunomodulation of the TME and for the enhancement of both ICIs and ACT.

## 2. NP-Based Delivery Systems for Cancer Therapy: An Overview

Nanocarriers can be developed to mimic the characteristics of immunogenic pathogens and provide tumor associated antigens (TAA) to re-establish and sustain the ongoing anti-tumor immune response in the TME [27,28]. Other strategies rely on the delivery of immunomodulatory drugs in the TME to modify the activity of tumor infiltrating lymphocytes [29]. In other cases, NP are utilized to deliver chemotherapeutics to the TME to specifically kill tumor cells, with consequent releases of TAA able to support anti-tumor immunity [30].

In terms of composition, NP can be divided into two main sub-categories: lipid-based or polymer-based NP [31,32]. Liposomes can, for example, be composed of bioinspired lipids and have a hydrophilic core that supports the loading of chemotherapeutics such as DOX [33], while polymer-based NP can be designed to have a hydrophobic core that can accommodate a vast variety of small hydrophobic molecules used in oncology. NP of these types can be composed of cationic lipids and polymers, designed to complex nucleic acids into the NP’s structure [34]. Other NP are protein-based and are designed to take advantage of the intrinsic “stealth” nature of biologically derived nanocages like ferritins [35]. NP can also be designed as hybrids between synthetic and bio-derived nanostructures. For example, cell-derived membranes can be used to coat lipid and polymeric NP to provide stealth and/or targeting capabilities [36].

One of the main advantages of NP-based delivery systems is the possibility of including in a single nanoformulation, multiple drugs which can have a synergistic effect. This led to the development of a multifaceted array of nanotherapeutics aimed at enhancing the ongoing anti-tumor immune response, leveraging one or more aspects of immunomodulation and immune stimulation.

The rationale for the design of nanocarriers is interdependent with the route of administration, the cellular target of choice and the therapeutic payload. The characteristics of NP have to be tailored to bypass specific physiological and intracellular barriers. For example, to reach the TME, NP are injected intravenously while nanovaccines are usually administered by intramuscular or subcutaneous injection.

In the next sections, we discuss the essential properties of NP to accomplish the delivery of therapeutics to the TME for immunomodulation and to induce anti-tumor immune responses in the case of nanovaccines.

### 2.1. Tailored Nanocarriers for the Delivery of Therapeutics to the TME

NP injected by the intravenous route are partially retained by the reticuloendothelial system (RES), composed of phagocytes such as circulating monocytes and tissue-resident macrophages [37]. These cells recognize NP as foreign objects and are able to effectively remove them from the circulation. To avoid recognition, NP can be coated with the hydrophilic polymer polyethylene glycol (PEG) which reduces protein adsorption and prolongs the half-life of NP in the bloodstream [38]. However, it has recently been shown that the immune system can react by producing anti-PEG antibodies, which could impact the use of PEGylated NP in the clinic [39]. PEGylation could reduce the effectiveness of NP after multiple administrations and limit their targeting capabilities, and could also induce undesirable immunogenic reactions. To avoid these issues, alternative strategies to reduce the recognition of NP by the immune system were recently developed. For example, zwitterionic polymers have been used to provide stealth capabilities to NP without inducing the production of antibodies [40,41]. Other strategies involve the coating of NP with CD47 moieties, which act as a potent “do not eat me” signal [42]. This strategy is adopted by pathogens such as vaccinia virus (smallpox), able to induce the expression of CD47 to escape recognition by RES [43]. In addition, NP can be coated with membranes derived from red blood cells or immune cells to mimic these cell types and improve tumor accumulation [44,45].

Interestingly, NP with a size of below 150nm passively accumulate in tumors via the Enhanced Permeability and Retention (EPR) effect [46]. The TME is characterized by blood vessels with abnormally wide fenestrations which allow for the extravasation and accumulation of NP particularly in the periphery of tumors. However, the extent of this pathophysiological phenomenon may vary between different tumors (even between primary and metastatic tumors), thus the accumulation of NP by passive targeting can be further enhanced by the addition of targeting ligands on their surface.

Receptors overexpressed by cancer cells or by other cells in the TME can be actively targeted by peptides, antibodies or other small molecules coupled to NP components [47]. For example, a number of different types of NP coated with Herceptin, an antibody that targets human epidermal growth factor receptor 2 (HER2), have been developed for the enhanced delivery of chemotherapeutics to HER2-positive tumors [48]. Interestingly, NP can also be coated with cancer cell-derived membranes providing homotypic targeting capabilities [49]. The rationale behind this strategy is based on the recognition of surface molecules on the NP coating by their cognate receptors expressed on the same population of tumor cells. Preclinical studies have shown the efficacy of this targeting strategy utilizing NP coated with membranes derived from 4T1 mammary carcinoma [50], HepG2 hepatocarcinoma [51], MCF-7 breast adenocarcinoma [52] and LNCaP-AI prostate carcinoma [53], among others. Active targeting can also be achieved by the coupling of peptides to NP components. Since peptides can be composed of a C-terminal carboxylic acid group, they can be coupled to amine-functionalized nanoparticles or vice versa [54,55]. A wide variety of peptides have been used to target overexpressed proteins in the TME [56,57]. For example, RGD peptides target integrins overexpressed in many tumors while the LyP-1 peptide targets p32, which is expressed mainly in breast cancer and TAMs [58,59]. Other widely used TME-targeting molecules and polymers include hyaluronic acid (HA) [60], mannose [61], folic acid [62] and transferrin [63].

NP can also be tailored to be stimuli-responsive to take advantage of the acid and hypoxic nature of the TME. This can be accomplished by incorporating stimuli-responsive compounds and polymers in nanostructures, with consequent release of their therapeutic payload in the TME [64,65,66]. Polymers composed of histidine, 4-vinyl pyridine, aspartic and methacrylic acid are some examples of pH-sensitive molecules widely included in NP formulations, while derivatives of nitrobenzil or azobenzene are incorporated as hypoxia-responsive elements [23]. In addition, NP can be made responsive to particular enzymes present in the TME such as metalloproteinases [67,68].

Even if NP are able to reach the TME, their internalization and entrapment in the endo-lysosomal compartments can lead to the partial degradation of their therapeutic cargoes, hampering their effectiveness. Initially, NP are entrapped in endosomes, which subsequently fuse with lysosomes, acidic vesicles loaded with numerous enzymes, able to digest internalized viruses and bacteria. In fact, many pathogens have evolved different strategies to evade, or take advantage of this process [69]. Cationic polymers, fusogenic peptides and other molecules derived from pathogens, can be incorporated in NP to induce endosomal release, and favor the accumulation of the therapeutic drugs in the cytoplasm of target cells, avoiding degradation [70]. Endosomal escape strategies include pore formation, membrane fusion, membrane destabilization or the proton sponge effect [70]. The incorporation of an endosomal escape molecular strategy in NP design is necessary to ensure the effective release of nucleic acids such as short interfering RNA (siRNA), micro RNA (miRNA), short hairpin RNA (shRNA) and DNA, which are particularly sensitive to endosomal degradation by RNAses and DNAses [34,71].

Overall, the physiological and intracellular barriers can be overcome by accurate NP design, ensuring enhancement of the pharmacokinetic properties of loaded drugs, protection from degradation, accumulation in the TME, and the reduction of off-target side effects.

### 2.2. NP-Mediated Immunomodulation of the TAMs

**Figure 1:**
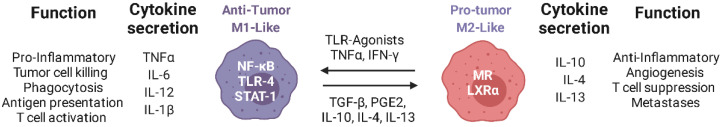
Schematic representation of the functions of M1-like and M2-like macrophages: Immunosuppressive molecules in the TME contribute to the polarization of TAMs towards an M2-like phenotype, with consequent support of tumor growth. Re-polarization of TAMs with TLR ligands and nanotherapeutics can promote their switch to an M1-like phenotype leading to the infiltration of activated cytotoxic T cells in the TME, ensuring the control of tumor growth. Created with Biorender.com.

In solid tumors, TAMs constitute up to 50% of the tumor mass [72]. They are recruited from the blood stream and surrounding tissues by chemokines and growth factors including C-C motif ligand 2 (CCL2), colony stimulating factor 1 (CSF-1) and vascular endothelial growth factor (VEGF) [73]. Macrophages have a plethora of transitional cell states and can be polarized in vitro towards two distinct phenotypes: M1 (pro-inflammatory) induced by lipopolysaccharide (LPS), interferon gamma (IFN-γ) and tumor necrosis factor alpha (TNF-α), and M2 (anti-inflammatory) induced by IL-4, IL-10, IL-13, prostaglandin E2 (PGE2) and transforming growth factor beta (TGF-β) [74]. Immunosuppressive molecules present in the TME are able to skew TAMs towards an M2-like phenotype and they primarily contribute to tumor growth by promoting angiogenesis and by limiting the effectiveness of TILs (Figure 1) [75,76]. In addition, it is known that high infiltration of TAMs is indicative of poor prognosis in many tumors [77]. On the other hand, the pro-inflammatory (M1-like) phenotype of macrophages is characterized by a high secretion levels of IL-12 and chemokine (C-X-C motif) ligands (CXCL) 9 and 10, crucial chemokines for the accumulation of T cells in the TME. Notably, CXCL9 and 10 were found to be upregulated in tumors after treatment with ICIs and were positively correlated with the degree of anti-tumor immunity [78].

Many nanotherapeutics have been developed to re-educate TAMs towards an M1-like phenotype, which is induced in vivo by molecules present on the surface of pathogens and their nucleic acids or by damage-associated molecular patterns (DAMPs) [79,80]. These molecules are sensed by pattern recognition receptors (PRRs), key mediators of the innate immune response present on the cellular and endosomal membranes of many cell types, in particular on phagocytes. Molecular sensors of this type include toll like receptors (TLRs), C-type lectin receptors (CLRs) and NOD-like receptors (NLRs). After binding to their cognate ligands, these receptors initiate the intracellular molecular cascade leading to the activation of the complement system and secretion of pro-inflammatory signals [81]. Interestingly, intratumoral injection of TLR ligands can induce strong anti-tumor immune responses supported by the activation and re-polarization of macrophages with consequent infiltration of activated CD8+ T cells in the TME [82,83]. However, this route of administration lacks the required translational potential since many human tumors are difficult to reach, and therefore to perform intratumoral injections would require invasive procedures.

To avoid this issue, NP were recently developed to re-educate TAMs towards an M1-like phenotype via the delivery of Poly I:C (TLR-3 ligand) entrapped in poly-arginine (PA) or poly-octarginine (PO) nanostructures coated with HA or polyglutamic acid-PEG (PGA-PEG) [84]. The results of this study showed that naked NP and HA-coated NP were avidly phagocytosed in vitro by macrophages, compared with PGA-PEG-coated NP. In addition, human macrophages treated with poly I:C-loaded NP were able to effectively kill pancreatic cancer cells (PANC-1) *in vitro*, similarly to M1-polarized macrophages [84]. In another study, the TLR-7/8 ligand resiquimod (R848) was encapsulated in cyclodextrin nanoparticles (R848-CDNP). These NP (size ~30 nm), were formed under aqueous conditions through amide bond formation between succinyl-β-cyclodextrin and L-lysine. Treatment of MC38 colon adenocarcinoma bearing mice with intravenous injections of R848-CDNP showed a reduction in tumor growth compared to controls, and evidence for re-programming of TAMs was demonstrated after only a single inoculation of R848-CDNP [85]. Wang and colleagues developed 1,2-dioleoyl-sn-glycero-3-phosphoethanolamine-mannose (DOPE-M) NP coated with O-carboxymethyl-chitosan (CMCS, a pH responsive polymer) for the delivery of IMD-034, a selective IκB-kinase β (IKKβ) inhibitor with M1-polarizing activity [86]. The intravenous coadministration of IMD-034-loaded NP with a nano formulation of the tyrosine kinase inhibitor, Sorafenib, showed reduced tumor growth in Hepa1-6 tumor-bearing mice compared to controls. Furthermore, in the TME of mice treated with a combination of the two nanosystems, the M1/M2 ratio of TAMs was higher, compared to free Sorafenib or encapsulated Sorafenib + free IMD-034 [86].

As explained in the previous section, NP delivery systems can be coated with different cell-derived membranes to bypass RES recognition and enhance the delivery of the therapeutic payload directly to the TME. By doing so, the coated NP can acquire some characteristics of the cell population of origin. Intriguingly, hybrid nanovesicles (HNVs) were devised to mimic M1-polarized macrophages and deliver the STING agonist cGAMP to the TME, to induce the re-education of TAMs toward an M1-like phenotype. Prior to coating, M1-derived membranes were fused with cell membranes derived from cancer cells and platelets to provide antigens and stealth capabilities to the generated HNVs. HNVs, with a size of ~100 nm, were tested on melanoma and breast cancer xenograft models (B16F10 and 4T1) where the primary tumor was partially removed to model post-surgery recurrence. The results of the study showed metastasis growth inhibition in both murine models treated after surgery with HNVs compared to controls and induction of CD8+ infiltration in the TME with enhanced secretion of IFN-γ [87]. In another report, Cao and colleagues developed novel NVs-like ginseng-derived nanoparticles (GDNP) isolated from Panax ginseng. Interestingly, these NVs are able to re-educate TAMs towards an M1-like phenotype by a mechanism dependent on TLR-4 activation. Mice challenged with B16F10 melanoma cells and treated with intraperitoneal injections of GDNP showed reduction of tumor growth, enhanced infiltration of CD8+ T cells and NK cells in the TME with evidence of re-polarization of TAMs. Of note, in vivo depletion of TAMs prior to GDNP administration dramatically reduced the treatment effectiveness of GDNP, showing that the re-polarization of TAMs plays a crucial role in the reduction of tumor burden in this xenograft model [88]. NVs are particularly alluring due to their reduced toxicity and high capability for customization. However, the production of NVs is demanding and has many bottlenecks: the lack of standardized isolation and purification methods, limited drug loading efficiency, and insufficient clinical grade production limit their translation potential [89].

Another therapeutic strategy to impact the immunosuppression of the TME relies on depletion of TAMs. This can be achieved by a liposomal formulation of the bisphosphonate clodronate (CodroLip) leading to tumor growth reduction in a wide variety of mouse xenograft models [90,91,92]. Of note, CodroLip therapy has been used in pre-clinical mouse models to study the effect of macrophage depletion in many diseases, including cancer [93]. However, this treatment can induce severe side effects such as neutrophilia and anemia, and it was not recommended for human trials due to its high toxicity [94]. Lastly, there is little interest from the pharmaceutical industry in the development of clodronate (or any other bisphosphonate) nano formulations, since these drugs are considered “old”, therefore non-patentable. Although CodroLip development was halted, many research groups have been actively working on the specific depletion of TAMs. The targeting of the CSFR-1 signaling pathway with small molecules inhibitors or antibodies showed evidence for the depletion of TAMs in pre-clinical studies, and clinical trials are currently ongoing [95]. NP can also be used to effectively deliver CSFR-1 targeting molecules to the TME. For example, Quian and colleagues developed a dual-targeted NP loaded with anti CSFR-1 siRNA to limit the accumulation of TAMs in the TME. Two peptides (ApoA 1-mimetic α-helical peptide and M2pep) were used to actively target TAMs, while the anti CSFR-1 siRNA was coupled to cholesterol and introduced in the NP membrane, composed of 1,2-dimyristoyl-sn-glycero-3-phosphocholine (DMPC) and 1,2-distearoyl-sn-glycero-3-phosphoethanolamine-PEG (DSPE-PEG). The administration of dual-targeted NP to B16 tumor bearing mice induced reduction in tumor growth, a fivefold increased infiltration of CD8+ T cells, and 50% reduction of TAMs, compared to controls [96].

Taken together, the experimental evidence supports the rationale for tumor targeting of TAMs to induce their re-education towards an M1-like phenotype, or to limit their infiltration in the TME. However, even if activated M1 macrophages are able to directly kill cancer cells, re-polarization of TAMs is insufficient to completely eradicate tumors in pre-clinical models. Immunomodulation of TAMs can be further supported by other immunotherapeutic strategies aimed at inducing a strong anti-tumor adaptive immune response. In the next section, we summarize recent advances in the field of anti-cancer nanovaccines that can be utilized to stimulate de-novo immune responses against TAA.

### 2.3. Nanovaccines for Cancer Therapy

Vaccines are constituted by two core components: antigen and adjuvant. After administration via intramuscular or subcutaneous injection, a vaccine’s components are taken-up by resident APCs, which then migrate to the lymph nodes to initiate the adaptive immune response. The adjuvant induces activation of APCs, and upregulation of costimulatory molecules on their surface, both of which are necessary to fully activate T cells and B cells resident in the lymph nodes. Anti-microbial vaccines are widely used, and are effective immunizing agents, able to induce protection against a large variety of pathogens. However, few anti-cancer vaccines are aimed at inducing protection, while the majority are utilized in therapeutic settings when the tumor is already present. In this case, the anti-tumor immune response has already failed in controlling tumor growth and the vaccine attempts to re-establish and strengthen the ongoing immune response. Unfortunately, the clinical translation of this approach has proven challenging, with only one candidate cancer vaccine reaching the clinical stage for prostate cancer [97,98].

**Figure 2:**
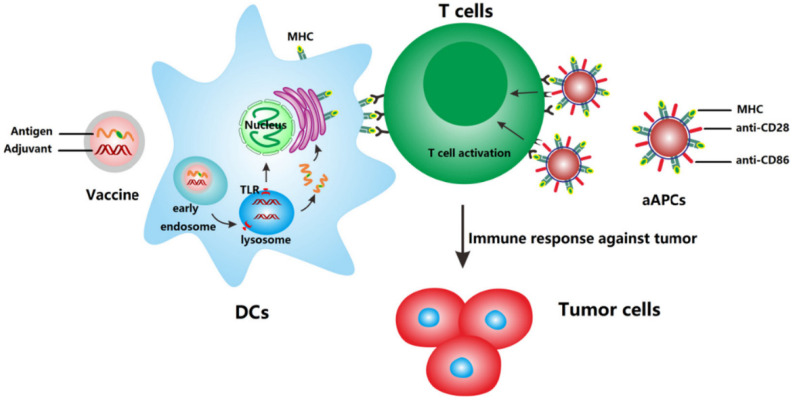
Mechanism of action of anti-cancer nanovaccines and artificial antigen presenting cells (aAPCs). Nanovaccines deliver antigens and adjuvants to DCs which subsequently upregulate co-stimulatory molecules and present the processed antigen to T cells via MHC molecules. aAPCs can directly activate T cells by MHC-bound antigens and costimulatory molecules present on cell-derived membranes on the surface of NP. Reprinted from [99] with permission from Elsevier.

NP-based delivery systems are an attractive tool for the development of anticancer vaccines, since they can co-deliver antigen and adjuvant simultaneously to the same cell (Figure 2).

In addition, NP can be tailored to extravasate into the lymphatic system to reach the lymph nodes directly. For this purpose, NP ranging from 10 to 100 nm have been shown to effectively reach lymph nodes after subcutaneous injection, while NP of a larger size are unable to drain effectively into the lymphatic system and are retained at the injection site [100,101]. The incorporation of antigens in NP can be achieved by covalent linkage of a protein or peptide to components of the nanostructure. In addition, nucleic acids can be attached through electrostatic interactions to the surface of NP (similarly to siRNAs) and can be processed and translated by APCs into antigenic peptides. Moreover, DNA and mRNA-based cancer vaccines can be designed to include multiple antigens to further increase immunogenicity. These nanosystems have the advantage of more closely mimicking live infections by incorporating multiple antigenic epitopes and pathogen-derived immune-adjuvants, into one single nanostructure.

Yang and colleagues developed poly (lactic-co-glycolic acid) (PLGA)-core/lipid-shell hybrid NP for the co-delivery of the adjuvant gardiquimod (TLR-7 ligand) and mRNA encoding for the model antigen ovalbumin (OVA). This formulation is composed of a hydrophobic PLGA core to entrap gardiquimod and a cationic liposome as particle coating to allow for mRNA adsorption by electrostatic interactions. The intravenous injection of the hybrid nanovaccine is able to induce growth reduction of B16-OVA xenograft tumors in prophylactic or therapeutic settings. In addition, co-stimulatory molecules on dendritic cells (DCs) were upregulated after the internalization of the hybrid nanovaccine, and antigen presentation on MHC molecules was enhanced, compared to the control nanovaccine without guardiquimod [102]. In another study, Liu and colleagues developed PLGA NP containing OVA coupled to MPGΔNLS, a cell-penetrating peptide, to enhance the release of OVA from the lysosomes and avoid antigen degradation. This formulation was superior to NP loaded with free OVA in inducing the secretion of TNF-α and IL-12 by bone marrow derived dendritic cells (BMDCs). In addition, the treatment of E·G7-OVA tumor bearing mice with intramuscular injection of PLGA MPG-OVA nanovaccine, was able to reduce tumor burden and enhance the infiltration of CD8+ T cells in the TME compared to controls [103].

To incorporate a wide variety of antigens into nanostructures, NP can be decorated with cell-derived membranes expressing TAA. For example, Cheng and colleagues developed IL-2-loaded PLGA NP entrapped in tumor cell lysate-pulsed BMDC-derived membranes (MiniDC). This methodology allows for the incorporation of tumor associated antigens onto the surface of BMDCs, which are then used as an envelope for IL-2-loaded PLGA NP. This synthetic nanovaccine mimics the activity of DCs and can efficiently present antigen and stimulate T cells, both in vitro and in vivo. Subcutaneous administration of MiniDC to murine xenografts of ovarian cancer cells (ID8 cells) promoted tumor growth inhibition and limited the development of metastasis after intraperitoneal injection of ID8 cells. However, in this case, the nanovaccine was administered prior to, and after the tumor challenge [104]. In a recent report, pH-dependent aAPCs were similarly developed to include IL-2, costimulatory CD28 and antigen loaded into MHC I molecules. These nano-sized aAPCs (naAPCs) have a diameter of 100 nm at physiological pH, which grows up to 1 µm in an acidic environment such as the TME. NaAPCs were developed to extravasate to the TME, and then enhance their size to be retained, and continuously stimulate activated CD8+ T cells. As proof of principle, mice bearing EG7-OVA tumors were either vaccinated with a nanoformulation of OVA (NP-OVA), treated with a nanoformulation of DOX (NP-DOX), or treated with a nanoformulation containing a photosensitizer (NP-HPHH) for photodynamic therapy. Subsequently, naAPCs loaded with the OVA-derived peptide SIINFEKL were introduced intravenously to enhance the capacity of the prior administered treatments to reduce tumor growth [105]. This interesting report shows that naAPCs can provide continuous intramural co-stimulation to T cells and can potentially synergize with a wide variety of other anti-tumor treatments.

Another methodology used to incorporate multiple antigens into NP relies on the coupling of the ubiquitin binding protein VX3 to NP, in order to bind ubiquitinated proteins (UPs) from tumor lysate extracts. A nanostructure based on α-Al2O3 was modified with VX3 proteins to allow the binding of UPs from 4T1 tumor cell lysate, to generate α-Al2O3-VX3-UPs. The treatment of 4T1 tumor bearing mice with α-Al2O3-VX3-Ups, injected subcutaneously, induced tumor growth reduction as a standalone treatment, and synergized with prior low-dose epirubicin to further improve its anti-tumor activity [106].

Both nanovaccines and aAPCs can effectively stimulate an anti-tumor immune response. However, their therapeutic efficacy could be further enhanced by ICIs, to remove immunosuppressive brakes in the TME, or by ACT, to provide a pool of activated T cells that can be re-stimulated in vivo by nanovaccines, to further increase their therapeutic potential.

## 3. NP-Based Delivery Systems Designed to Improve ICI and ACT Immunotherapies

Immunotherapeutic strategies rely on the infiltration of activated CD8+ T cell in the TME to kill cancer cells. However, when the TME is highly immunosuppressive, T cells are unable to efficiently exert their function and can become anergic. Thus, acting on the immunosuppressive TME, while at the same time inducing a strong a specific anti-tumor immune response, is essential to achieve a strong durable response able to eradicate established tumors. In the next section, we will discuss recent nanotherapeutic approaches developed in the last five years aimed at enhancing ICIs and T cell-based ACT (summarized in Table 1). In Table 2, we summarize the main functions of NP-based therapeutics that can synergize with ICIs and/or ACT.

### 3.1. Nano-Therapies Enhancing ICIs

Therapeutic nanovaccines have been recently employed to stimulate a de novo immune response against tumor neoantigens. However, as a standalone therapy, nanovaccines are not able to completely eradicate established tumors. To improve the efficacy of nanotherapeutics, many research groups have explored their use in combination with ICIs in pre-clinical models with great success. For example, Luo and colleagues developed a pH-sensitive nanostructure based on the polymer PC7A, which provides STING-activating properties, loaded with different TAA. The effectiveness of this nanotherapeutic vaccine was dramatically enhanced as a combination therapy with an anti-PD-1 antibody, leading to more than 50% survival in B16-OVA xenograft, and 100% survival over 60 days in a TC-1 tumor model [107]. In another report, Shae and colleagues developed pH-responsive polymeric NP comprised of endosomolytic diblock polymers loaded with peptide antigens and the STING agonist, cGAMP (nanoSTINGvax). This nanoformulation, in combination with an anti-PD-1 antibody, was able to induce tumor rejection in 80% of mice previously implanted with MC38 tumors [108]. Another two-in-one polymeric nanosystem was developed to co-deliver the STING agonist, DMXAA, and SN38 (an irinotecan pro-drug) to induce an immunogenic TME. The SN38-prodrug building block serves as a hydrophobic core during NP self-assembly. The pro-drug is then cleaved off from the polymer in response to the redox stimuli in tumors. In B16F10 melanoma xenografts, this formulation was able to reduce tumor growth and improve the efficacy of PD-1 blockade [109]. Similar results were obtained by PEGylated reduced graphene oxide nanosheet nanovaccine, in combination with anti PD-1 therapy [110]. Ferritin nanocages have also recently been used as cancer vaccine platforms. These types of NP are composed of ferritin, an iron-storage protein, which autonomously self-assembles as 24-mer nanocages of 12 nm. Wang and colleagues showed that a ferritin-based nanovaccine synergizes with anti PD-1 therapy in reducing tumor burden in the MC38 colon cancer xenograft model [111]. Compelling evidence of the nanovaccine’s efficacy in combination with ICIs was recently shown in the Lipo-MERIT trial, where an RNA-based liposomal nanovaccine, loaded with four different melanoma antigens (FixVac), demonstrated efficacy in patients with advanced melanoma, who had progressed after PD-1 therapy [112]. Intriguingly, FixVac was able to partially re-sensitize patients to the PD-1 blockade.

**Figure 3:**
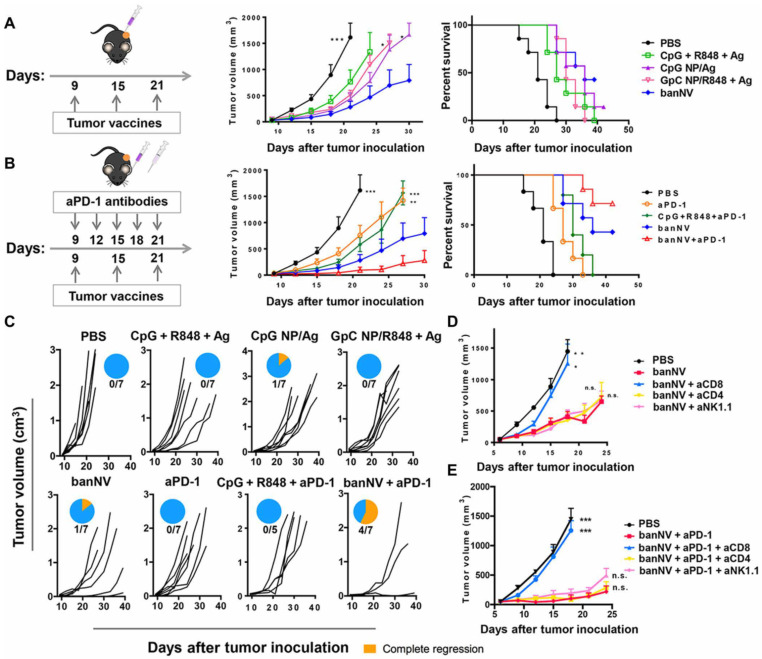
Combination of banNVs with immune checkpoint blockade markedly promotes response and complete regression of MC38 tumors in syngeneic mice: (**A**) Left: Experimental design for tumor immunotherapy in C57BL/6 mice with the indicated formulations of vaccines; middle: tumor growth curve, and right: mouse survival of C57BL/6 mice after subcutaneous inoculation with MC38 tumor cells. (**B**) Left: Experimental design for combination tumor immunotherapy in C57BL/6 mice with banNVs and anti PD-1; middle: tumor growth curve; and right: mouse survival of C57BL/6 mice after subcutaneous inoculation with MC38 tumor cells. (**C**) Individual tumor growth and survival profiles of C57BL/6 mice treated with vaccines and/or anti PD-1 over 40 days. (**D**,**E**) Tumor growth curve after vaccination with banNVs or the combination of banNV and anti PD-1, together with lymphocyte depletion by anti-CD8, anti-CD4 or anti-NK1.1. Reproduced from reference [113] without changes. * *p* < 0.05, ** *p* < 0.01, and *** *p* < 0.001.

The combination of multiple TLR ligands is able to enhance the efficacy of nanovaccines by inducing strong activation of APCs leading to tumor eradication of MC38 tumor xenografts, dramatically improving anti-PD-1 therapy. R848 and CpG (TLR 7/8 and TLR9 ligands) were co-encapsulated with the peptide antigen, ADPGK (banNV), and showed enhanced APCs function and tumor growth control compared with NP loaded with only one TLR ligand. The efficacy of the dual nanovaccines was further enhanced by anti-PD-1 therapy, leading to complete tumor regression in 57% of treated mice (Figure 3) [113]. In another report, mannosylated polylactic-co-glycolic acid (PLGA)/polyl(l-lactic acid) (PLA) (man-NP) was developed to incorporate melanoma TAA plus CpG and monophosphoryl lipid A (MPLA, a TLR-4 ligand). This nanosystem was used in combination with anti-PD-1 therapy and OX-40 antibodies, and exhibited strong synergistic effect in controlling tumor growth in xenografts models. The authors reported that treatment with man-NP induced accumulation of MDSCs in the TME, limiting anti-tumor efficacy. However, inclusion of ibrutinib in the therapeutic protocol was able to reduce this detrimental effect [114]. Of note, it has been shown by others that treatment with ibrutinib inhibits breast cancer progression and metastasis, by inducing conversion of myeloid-derived suppressor cells to dendritic cells [137,138].

Interestingly, nanoparticles can be tailored to effectively bypass lysosomal degradation to release antigens in the cytoplasm of APCs, while inducing their activation. Gong and colleagues developed a nanotransformer-based vaccine (NTV) composed of the adjuvant, CpG, along with TAA. In acidic media, NTV transforms into larger structures, which causes endosomal membrane disruption and cytosolic delivery of the loaded antigen. NTV was successfully used in combination with anti PD-L1 antibodies leading to B16F10 tumor eradication in 50% of treated mice, while the treatment with NTV or anti-PD-L1 alone, was only able to delay tumor growth [115].

In the vast majority of studies involving nanotherapeutics in combination with ICIs, both treatments are administered during the same time window. Recently, Kim and colleagues showed that anti PD-1 therapy administered 1 week after the last immunization with a CpG-based nanovaccine was more effective in controlling tumor growth compared to administration between vaccinations. This study suggests that the removal of the immunosuppressive blockade with anti-PD-1 antibodies is less effective if there is a sub-optimal ongoing T cell response, and provides novel insight for the development of therapeutic protocols with other immunotherapy modalities [116].

The choice of TAA is a critical step in the development of nanovaccines. In fact, the impressive results obtained in pre-clinical models are mediated by the targeting of specific antigens, which are, in some cases, model antigens such as OVA. In these cases, the model antigen is overexpressed by tumor cells and cannot be considered as a surrogate of a mutated or overexpressed self-antigen present in human tumors. To circumvent this problem, Xu and colleagues developed a nanoplatform able to include neoantigens from the surface of tumor cells. This strategy allows the integration of tumor extract from resected autologous tumors with a fluoropolymer-based NP and can effectively prevent post-operative tumor recurrence and tumor metastases in treated mice, if used in combination with anti PD-1 or anti CTLA-4 therapy [117].

The efficacy of ICIs is limited by the low accumulation of these antibodies in the inner core of solid tumors. To enhance ICI penetration, Jiang and colleagues developed a nanoplatform based on the polymer HA, able to target tumor endothelial cells, to deliver apatinib (a VGFR2 inhibitor) together with lonidamine. Apatinib provides vascular normalization by decreasing interstitial fluid pressure [139] while lonidamine inhibits lactic acid efflux in the TME to enhance T cell function. The combination with anti PD-1 therapy induced reduction of melanoma tumor growth, and enhancement of T cell infiltration, while limiting Tregs accumulation in the TME [118]. Interestingly, the increase in efficacy of anti PD-1 therapy as a result of limiting the lactic acid efflux in the TME, has been recently reported by other groups, who have developed NP to deliver the MCT1 inhibitor, AZD3965, anti LDHA or VEGFR2 siRNAs [119,120,121].

One probable cause of the limited efficacy of ICIs in solid tumors is the presence of immunosuppressive cells (TAMs and MDSCs), which are able to limit T cell responses by secreting immunomodulating factors in the TME. Hence, the blockade of multiple immunosuppressive pathways simultaneously could further increase active T cell infiltration in the TME, with consequent tumor control. Yang and colleagues developed a functional nanomaterial, layered double hydroxides (LDHs), loaded with miR155, which is able to re-polarize TAMs. This nanoformulation, used in combination with anti PD-1 therapy, was able to reduce tumor growth by skewing TAMs towards an M1-like phenotype while, at the same time, limiting accumulation of MDSCs in the TME [122]. In another report, PD-1-targeted NP loaded with R848 synergized with anti-PD-1 therapy in reducing tumor growth of MC38 xenografts [123].

Innate immune cells stimulated with PRR ligands undergo metabolic and epigenetic rewiring and adjust their functional programs in a process termed ‘‘trained immunity”. This type of myeloid re-programming can be considered a de facto innate immune memory [140]. The induction of trained immunity by bone marrow-targeted HDL-nanodiscs loaded with a muramyl dipeptide derivative, showed a synergistic effect with ICIs in controlling B16F10 tumor growth. This approach was aimed at shifting the TME to a pro-inflammatory anti-tumor state by re-programming and enhancing myelopoiesis. The administration of the immunomodulating nanodiscs led to enhanced antigen presentation and cytokine secretion by APCs, which support the anti-tumor T cell function in the TME. In addition, this study showed that the developed nanodiscs displayed a favorable safety profile, paving the way for clinical translation [124].

Unfortunately, therapy with ICIs can give rise to immune-related side effects in patients leading to treatment discontinuation and, in some cases, serious autoimmune events [141]. To overcome this problem, many research groups have developed nanotherapeutics able to deliver ICIs specifically to the TME in order to reduce off-site side effects. For example, anti-PD-L1 siRNAs were co-encapsulated with DOX in polymer-lipid hybrid nanovesicles [125] or co-encapsulated with the TGF-β inhibitor LY2157299 [126]. In another study, poly(β-L-malic acid) (PMLA) NP were used to facilitate the delivery of CTLA-4 and PD-1 antibodies to brain tumors [127]. NP were also developed to deliver the small molecule BMS-202, an inhibitor of the PD-1/PD-L1 interaction. Interestingly, these NP were co-loaded with Ce6, a photosensitizer, which has been widely exploited for photodynamic therapy (PDT) [128]. However, PDT is usually used to treat tumors on or just under the skin or on the lining of internal organs or cavities [142].

There is an ever-increasing quantity of pre-clinical evidence in support of combinatorial treatment with ICIs and nanotherapeutics. We hypothesize that, in the near future, implementation of NP-based treatments devised to support ICIs will translate into the clinical setting to dramatically increase the number of patients that can benefit from immunotherapies.

### 3.2. Nanotherapies Enhancing T Cell-Based ACT

Adoptive cell therapy protocols based on T cells, in particular CAR-T cells, have been used effectively to treat a wide variety of hematological cancers. However, their production requires ex vivo manipulation, expansion and subsequent reimplantation. To reach a clinically meaningful number of T cells, the expansion phase requires long periods of time, leading to delays in the treatment schedule and high costs of production. In order to reduce both the production time and the costs involved, Smith and colleagues developed a DNA-carrying NP able to transduce CAR genes into T cell nuclei in vivo. These NP, targeted with an anti CD3 antibody, were composed of a peptide containing microtubule-associated sequence (MTAS) and a nuclear localization signaling (NLS), to facilitate the nuclear delivery of the co-encapsulated DNA cargo, composed of the CAR sequence, combined with 4-1BB and the CD3ζ cytoplasmic signaling domain. The generation of CAR-T cells in vivo after NP injection was able to achieve impressive results in a leukemia mouse model, comparable to implanted CAR-T cells produced ex vivo [129]. In another report, Parayath and colleagues have developed a similar NP-based strategy to transiently induce CAR expression on T cells in vivo. In this case, an mRNA transcript encoding the CAR gene, which does not require nuclear delivery, was condensed to the cationic polymer PBAE-447 to form NP targeted to CD8+ T cells. Interestingly, these NP were effective in mouse models of human leukemia, prostate cancer and hepatitis B–induced hepatocellular carcinoma with comparable results to re-infused ex vivo engineered CAR-T cells. A Phase I clinical trial to treat patients with HBV-related hepatocellular carcinoma is currently ongoing [130]. This strategy could potentially be applied for the in vivo generation of CAR-T cells specific for solid tumors.

Unfortunately, the clinical translation of CAR-T cells for the treatment of solid tumors showed only moderate success in clinical trials [143,144]. This is, in part, due to the low infiltration of the infused T cells in the TME, which then encounter multiple immunosuppressive signals able to reduce their anti-tumor function. To further stimulate the expansion and effectiveness of transduced T cells in vivo, different NP-based “backpack” strategies have been developed to deliver immunomodulating agents together with T cells in the TME. Protein nanogels targeted to CD45, which served as a stable, non-internalizing anchor, were employed to bind to T cells and slowly release an IL-15 superagonist complex in the TME to support T cell effector functions. This strategy improved the efficacy of CAR-T cells in B16F10 xenografts dramatically, leading to complete tumor eradication in 80% of treated mice, compared to only 20% in mice treated with standard CAR-T cells [131]. In another report, CD45-targeted PEGylated immunoliposomes loaded with a TGF-β inhibitor were used as a backpack prior to CAR-T cell infusion, leading to enhanced T cell efficacy compared to controls. Interestingly, subsequent injection of immunoliposomes after T cell transfusion led to a further enhancement of the transfused T cells, with consequent tumor growth control in B16F10 xenografts [132]. Another type of cross-linked, multilamellar liposomal vesicles (cMLV) backpack for CAR-T cells was developed to deliver the A2a adenosine receptor (A2aR) antagonist, SCH-58261. Adenosine in the TME suppresses T cell proliferation and IFN-γ secretion. Therefore, the blockade of this molecular pathway in infiltrating T cells improved their tumor-killing capacity [133]. T cell backpacks can also be used to deliver cytotoxic drugs in the TME to slowly release the cytotoxic agent directly into the tumor milieu, enhancing its effectiveness while reducing off-target effects. Kim and colleagues developed a novel click-chemistry-based methodology to couple NP to CAR-T cells prior to infusion. In this case, pH-sensitive NP were loaded with DOX and the treatment of glioblastoma-bearing mice with backpacked CAR-T cells showed increased tumor accumulation of DOX, compared to the free drug [134].

The efficacy of CAR-T cells can also be improved by the treatment with immunomodulatory NP prior to T cell transfusion. This strategy can support CAR-T cells homing to the tumor lesion, leading to an enhanced expansion and anti-tumor function in the TME. For example, 4T1-ROR1 tumor bearing mice treated with an integrin-targeted liposomes loaded with a combination of the PI3K inhibitor PI-3065, and the α-GalCer agonist 7DW8-5, showed enhanced efficacy of transplanted CAR-T cells which were able to eradicate tumors in 50% of treated mice, while NP and CAR-T cells alone were ineffective [135].

In another interesting report, an mRNA liposomal nanovaccine, RNA-LPX, was developed to deliver the CAR target to lymphoid tissues to support the expansion of previously infused CAR-T cells. In this report, RNA-LPX cationic liposomes based on DOPE and DOTMA were used to protect and deliver mRNAs containing the CAR target sequence (CD19 or CDLN6) and were administered to support the in vivo efficacy of CD19-, CLDN6- and CLDN18.2-targeted CAR-T cells, respectively. Treatment with RNA-LPX was able to strongly enhance the in vivo expansion of previously transplanted T cells, which showed an effector memory and a central memory phenotype. In addition, RNA-LPX treatment did not induce cytokine release syndrome, or depletion of APCs in the lymphoid tissues, and supported therapeutic tumor control in different murine tumor models mediated by a sub-therapeutic dose of infused CAR-T cells [136].

These recent reports highlight multiple strategies that can be used to augment the efficacy of CAR-T cells by improving their tumor-homing and by slowly releasing immunomodulating or cytotoxic drugs directly in the TME to support T cell function. Furthermore, nanovaccines can be used to support in vivo T cell proliferation, to provide a strong, sustained activity of the implanted CAR-T cells to treat solid tumors.

## 4. Drawbacks and Future Perspectives

In general, NP-based therapeutics show strong anti-tumor effects in pre-clinical models of cancer. Nonetheless, clinical trials have provided little evidence of efficacy, especially if NP are administered as a standalone treatment. This could be explained by the exaggerated intratumoral distribution of NP in xenograft models, which harbor a relatively well-developed tumor vasculature, enhancing the passive targeting of NP mediated by the EPR effect. Murine xenograft models are rapidly proliferating, in addition to being highly vascularized tumors, which is also very different from their human counterparts, characterized by a more complex stromal architecture and a higher stromal density. Therefore, NP-based therapeutics, which are effective in murine models of cancer, may encounter additional issues to achieve similar results in human solid tumors. Slow growing tumor models or transgenic mice models could be employed to better recapitulate the stromal architecture of human tumors, since NP accumulation can dramatically differ between patients due to intrinsic heterogeneity [145].

In a meta-analysis study, which compared the efficacy of free DOX to liposomal formulations loaded with DOX in numerous clinical trials, the authors concluded that NP delivery did not significantly improve the efficacy of the therapeutic drug compared to administration in its free form [146]. However, NP-based therapeutics have a different spectrum of side effects, which is favorable, particularly in the case of cardiotoxic drugs such as DOX [147]. In addition, two review studies from 2016 have highlighted how NP-based therapeutics injected intravenously do not extravasate to tumors, and effectively only reach between 0.1 and 10% of the injected dose in the TME, both in animal models and patients [148,149]. Therefore, the strong claim that NP-based therapeutics can efficiently target tumor cells has been shown to be partly wrong, since the vast majority of the injected nanodrugs are rapidly cleared before reaching the TME. In contrast, NP physicochemical properties are very heterogenous, and specific considerations should be taken for each nanotherapeutic, because in some cases (e.g., Doxil™, Abraxane™, Marqibo™, DaunoXome™, and others), NP-based delivery could improve the therapeutic potential of the loaded drug and ameliorate its side effects [25].

Ouyang and colleagues have suggested that a specific dose threshold of NP could be necessary to achieve correct tumor targeting to enhance the effectiveness of treatment. They showed that a dose higher than 1.5 trillion NP/mouse (1 quadrillion in humans) is sufficient to achieve good tumor targeting, while lower doses of NP are quickly cleared by the RES, and are not able to achieve sufficient drug concentration in the TME. This study advances the hypothesis that prior injection of blank NP could be employed to saturate the non-specific clearance of NP, while a subsequent therapeutic injection could achieve better tumor targeting, with a consequent increase of the therapeutic effect of the loaded drug [150]. Another recent study challenged the status quo of the EPR effect as the primary mechanism of NP infiltration in the TME. The authors showed that gold NP with different core sizes (10, 50 and 100 nm) extravasate in the tumor milieu primarily by an active process through endothelial cells surrounding the blood vessels, rather than from passive accumulation mediated by gaps in the tumor vasculature [151]. These studies therefore show how the field of nanobiotechnology has not yet reached full maturity, since outcomes from different experimental approaches seem to contradict each other. Comparing results from different studies is particularly challenging in this field due to considerable heterogeneity between different nanoformulations developed that are not always comparable, leading to results that are co-dependent with the physicochemical properties and composition of the singular NP proposed.

Interestingly, cancer nanovaccines do not have to infiltrate the TME to fulfill their immunostimulatory role, thus the drawbacks previously mentioned should only have a minor impact on these types of therapies. In 2015, Carl Fidgor designed the PRECIOUS project, which was funded through the Horizon2020 European Program to support research aimed at scaling-up biodegradable nanomedicines for multimodal precision cancer immunotherapy. The consortium has been working on two types of GMP biodegradable PLGA nanoparticles: The first is a nanovaccine, containing tumor antigens and the immune activator α-GalCer analog IMM60 [152], and secondly, a NP embedded with compounds that could reverse the suppression and reactivate immunity in the tumor milieu. The true final goal of this project is to translate the preclinical findings into a Phase I clinical trial (ClinicalTrials.gov Identifier NCT04751786) testing safety and immunological efficacy of the developed non-liposomal nanomedicines for cancer immunotherapy.

There is a strong rationale for the use of cancer nanovaccines in combination with other immunotherapies or immunomodulators. NP offer the optimal platform for combinational immunotherapy, as they are able to encapsulate multiple immunomodulators and/or neoantigens in biodegradable particles. The main argument for this combinatorial therapeutic approach relies on the reinvigoration of the adaptive immune response against tumor cells by the nanovaccines with a simultaneous and synergistic treatment aimed at reducing the local immunosuppression in the TME. Furthermore, pre-clinical evidence also supports this hypothesis for CAR-T cell therapy, where nanovaccines can be utilized to enhance the in vivo expansion of transplanted T cells to augment and prolong their anti-tumor activity [136]. In addition, NP-based therapeutic strategies aimed at activating, re-polarizing or depleting the myeloid cellular compartment within the TME have shown compelling evidence for synergy with both ICIs and CAR-T cells. Therefore, translation of NP-based therapeutics should be accompanied in clinical trials in combination with other immunotherapies or immunostimulators to attack advanced metastatic tumors from multiple and different angles, in order to limit the strong immunosuppressive role of the TME, and support cytotoxic T cell functions.

## Figures and Tables

**Table 1:** NP-based delivery systems designed to improve ICI and ACT immunotherapies.

Delivery Platform	Composition	Support Therapy	Cancer Model	Reference
Polymeric NP	PEG-b-PC7A, neoantigens	αPD-1	B16OVA, E6/7 TC-1	[107]
Polymeric NP	PEG-DBP, cGAMP, neoantigens	αPD-1 + αCTLA-4	MC38	[108]
Polymeric NP	PEG-b-PSN38-b-PDEA, DMXAA	αPD-1	4T1, B16F10	[109]
Reduced graphene oxide nanosheet	RGO-PEG, CpG, neoantigens	αPD-1	B16.F10	[110]
Ferritin Nanocage	Modified *Pf* ferritin, neo-antigens	αPD-1	MC38	[111]
Liposome	R-DOTMA, DOPE, neoantigens	αPD-1	Advanced Melanoma	[112]
Coated nanomicelle	PEG-PLA, PPT-g- PEG, CpG, R848, Adpgk peptide	αPD-1	MC38	[113]
Polymeric NP	Man-PLGA, PLA, CpG, MPLA, neoantigens	αPD-1 + αOX40 + Ibrutinib	Ret melanoma, B16F10	[114]
Polymer-peptide NP	OEGMA-MAEMA-MAVE-NDP, neoantigens	αPD-L1	B16F10	[115]
Polymeric NP	DOPE, DSPE-PEG, MA-Chol, CpG, neoantigens	αPD-1	E.G7	[116]
Fuoropolymeric NP	F_13_-PEI, neoantigens	αPD-1 or αCTLA-4	B16F10, CT26, 4T1	[117]
Jet-lagged NP	mPEG-PLA, Chitosan, Apatinib, Lonidamine, HA, PSS	αPD-1	B16F10	[118]
UPS micelle NP	PEG-b-(poly(dipropylaminoethylmethacrylate), AZD3965	αPD-1	TC1	[119]
Polymeric NP	PEG-PLA, DOTAP, siLDHA	αPD-1	B16F10, 4T1	[120]
Polymeric NP	RGD-PEG-DSPE, ssPalmO-Phe, Chol, siVEGFR2	αPD-1	MC38	[121]
Layered double hydroxides (LDH) NP	LDH, miR155	αPD-1	TC1	[122]
PLGA-based NP	PLGA, PD-1-PEG-PLGA, R848	αPD-1	MC38	[123]
HDL-based nanodisc	ApoA1, DMPC, Chol, MTP	αPD-1 or αCTLA-4	B16F10	[124]
Polymer-lipid hybrid NV	PEAD, PC, Chol, siPD-L1, DOX	αPD-L1 siRNA in NP	B16	[125]
Polymeric NP	PEG-PCL, PCL, PCL-CDM-PAMAM, LY2157299, siPD-L1	αPD-L1 siRNA in NP	Panc02	[126]
Polymeric NP	PMLA, mPEG5000, a-msTfR, αPD-1 or αCTLA-4	αPD-1 and/or αCTLA-4 in NP	GL261	[127]
Self-assembled NP	BMS-202 (PD-1/PD-L1 inhibitor) and/or Ce6	αPD-L1	4T1	[128]
Polymeric NP	PGA, PBAE, CAR plasmid (DNA)	delivery of CAR in vivo	Eμ-ALL01	[129]
Polymeric NP	PGA, PBAE, CAR mRNA	delivery of CAR in vivo	Eμ-ALL01, LNCap C42, HepG2	[130]
Protein nanogel backpack	NHS-SS-NHS or NH2-PEG10k-NH2, ALT-803	pmel-1 Thy1.1+ CD8+ T cells	B16F10	[131]
Immunoliposome backpack	PEG-DSPE, Chol, HSPC, SB525334	pmel-1 Thy1.1+ CD8+ T cells	B16F10	[132]
Multilamellar liposomal vesicles backpack	DOPC, DOPG, MPB-PE, mPEG-SHSCH-58261	CD19 targeted CAR-T cells	SKOV3.CD19	[133]
Clickable polymeric NP backpack	BPLP-PLA, DOX	IL13 targeted CAR-T cells	U87Luc	[134]
Liposome	PC, Chol, PEG, DSPE-PEG, PI-3065, 7DW8-5	ROR1 targeted CAR-T cells	4T1-ROR1	[135]
Liposome	DOPE, DOTMA, mRNA	CLDN6, CD19 and CLDN18.2 targeted CAR-T cells	various	[136]

**Table 2:** Main functions of nanotherapeutics in synergy with immunotherapies.

NP-Based Therapeutics	Function in Synergism with ICIs and ACT
Nanovaccines, aAPCs	Stimulate adaptive anti-tumor immune responses Enhance T cell infiltration in the TME Sustain CAR-T cells proliferation and efficacy
Nano immunomodulators	Stimulate both innate and adaptive immunity Polarize TAMs towards an M1-like phenotype Enhance T cell infiltration in the TME Depletion of MDSC and Tregs in the TME
Nano chemotherapeutics	Directly kill tumor cells with consequent release of neoantigens and stimulation of the immune response
Nano backpacks	Enhance homing and function of CAR-T cells

## Abbreviations

A2aR	A2a adenosine receptor
aAPCs	artificial antigen presenting cells
ACT	adoptive cellular transfer
APCs	antigen presenting cells
BMDCs	bone marrow derived dendritic cells
CAR-T	chimeric antigen receptors T cells
CCL2	C-C motif ligand 2
CLRs	C-type lectin receptors
CMCS	O-carboxymethyl-chitosan
cMLV	multilamellar liposomal vesicles
CSF-1	colony stimulating factor 1
CTLA-4	cytotoxic T-lymphocyte antigen-4
CXCL	chemokine (C-X-C motif) ligands
DAMPs	damage-associated molecular patterns
DCs	dendritic cells
DMPC	1,2-dimyristoyl-sn-glycero-3-phosphocholine
DOPE-M	1,2-dioleoyl-sn-glycero-3-phosphoethanolamine-mannose
DOX	doxorubicin
DSPE-PEG	1,2-distearoyl-sn-glycero-3-phosphoethanolamine-PEG
EPR	enhanced permeability and retention
GDNP	ginseng-derived nanoparticles
HA	hyaluronic acid
HER2	human epidermal growth factor receptor 2
HNVs	hybrid nanovescicles
ICIs	immune checkpoint inhibitors
IFN-γ	interferon gamma
IKKβ	IκB-kinase β
DOX	doxorubicin
LDHs	layered double hydroxides
LPS	lipopolysaccharide
M1	pro-inflammatory macrophage
M2	anti-inflammatory macrophage
MDSCs	myeloid derived suppressor cells
MHC	major histocompatibility complex
miRNA	micro RNA
MTAS	microtubule-associated sequence
NK	natural killer cells
NLRs	NOD-like receptors
NLS	nuclear localization signaling
NP	nanoparticles
NSCLC	non small-cell lung carcinoma
OVA	ovalbumin
PA	poly-arginine
PD-1	programmed cell death protein 1
PDT	photodynamic therapy
PEG	polyethylene glycol
PGA-PEG	poly glutamic acid-PEG
PGE2	prostaglandin E2
PLA	polyl(l-lactic acid)
PLGA	poly (lactic-co-glycolic acid)
PMLA	poly(β-L-malic acid)
PO	poly-octarginine
R848	resiquimod
RES	reticuloendothelial system
scFv	single chain variable fragment
shRNA	short hairpin RNA
siRNA	short interfering RNA
TAA	tumor associated antigens
TAMs	tumor associated macrophages
TCR	T cell receptor
TGF-β	transforming growth factor beta
TILs	tumor-infiltrating lymphocytes
TLRs	toll like receptors
TME	tumor microenvironment
TNF-α	tumor necrosis factor alpha
Tregs	T regulatory cells
UPs	ubiquitinated proteins
VEGF	vascular endothelial growth factor
